# Rethinking counselling in prenatal screening: An ethical analysis of informed consent in the context of non‐invasive prenatal testing (NIPT)

**DOI:** 10.1111/bioe.12760

**Published:** 2020-07-04

**Authors:** Adriana Kater‐Kuipers, Inez D. de Beaufort, Robert‐Jan H. Galjaard, Eline M. Bunnik

**Affiliations:** ^1^ Department of Medical Ethics and Philosophy of Medicine Erasmus MC, University Medical Centre Rotterdam Rotterdam The Netherlands; ^2^ Department of Clinical Genetics Erasmus MC, University Medical Centre Rotterdam Rotterdam The Netherlands

**Keywords:** counselling, informed choice, informed consent, non‐invasive prenatal test, prenatal screening, reproductive autonomy, stepwise counselling model

## Abstract

Informed consent is a key condition for prenatal screening programmes to reach their aim of promoting reproductive autonomy. Reaching this aim is currently being challenged with the introduction of non‐invasive prenatal testing (NIPT) in first‐trimester prenatal screening programmes: amongst others its procedural ease—it only requires a blood draw and reaches high levels of reliability—might hinder women’s understanding that they should make a personal, informed decision about screening. We offer arguments for a renewed recognition and use of informed consent compared to informed choice, and for a focus on value‐consistent choices and personalized informational preferences. We argue for a three‐step counselling model in which three decision moments are distinguished and differently addressed: (1) professionals explore women’s values concerning whether and why they wish to know whether their baby has a genetic disorder; (2) women receive layered medical‐technical information and are asked to make a decision about screening; (3) during post‐test counselling, women are supported in decision‐making about the continuation or termination of their pregnancy. This model might also be applicable in other fields of genetic (pre‐test) counselling, where techniques for expanding genome analysis and burdensome test‐outcomes challenge counselling of patients.

## 1 INTRODUCTION

In many countries, when a pregnant woman first visits an obstetric care provider, she will be offered information about several prenatal screening tests. Some tests are offered to promote the health of mother or child, for example screening for Rhesus factor. Other prenatal screening tests, however, are aimed at the detection of foetal abnormalities for which no therapeutic or preventive interventions are possible or available.^1^Holland, W. W., Stewart, S., & Cristina, M. (2006). *Policy brief screening in Europe*. World Health Organization on behalf of The European Observatory on Health Systems and Policies. Copenhagen: WHO Regional Office for Europe. Rather, testing for these foetal abnormalities provides reproductive options to pregnant women or couples, with the aim of promoting reproductive autonomy.^2^Dondorp, W., De Wert, G., Bombard, Y., Bianchi, D. W., Bergmann, C., Borry, P., … Cornel, M. C. (2015). Non‐invasive prenatal testing for aneuploidy and beyond: Challenges of responsible innovation in prenatal screening. *European Journal of Human Genetics, 23*(11), 1438–1450. These tests enable future parents (a) to obtain information about their future child, and (b) decide about whether to continue or terminate a pregnancy in case of a genetic disorder.

Non‐invasive prenatal testing (NIPT) is being introduced widely as a screening test for three common foetal aneuploidies: trisomy 21, 18 and 13, leading to Down’s, Edwards’ and Patau’s syndrome, respectively. NIPT is an alternative for and an improvement of the first‐trimester combined biochemical test for these trisomies.^3^Oepkes, D., Page‐Christiaens, G. C., Bax, C. J., Bekker, M. N., Bilardo, C. M., Boon, E. M. J., … Sistermans, E. A. (2016). Trial by Dutch laboratories for evaluation of non‐invasive prenatal testing. Part I—clinical impact. *Prenatal Diagnosis, 36*(12), 1083–1090. It is based on the assessment of cell‐free DNA in the blood of the mother and has better test characteristics compared to the first‐trimester combined test, being more accurate and reliable. However, these advantages of NIPT have raised several ethical questions and concerns.^4^Nuffield Council on Bioethics. (2017). *Non‐invasive prenatal testing: Ethical issues*. London, UK: Nuffield Council on Bioethics. For instance, an increase in uptake of NIPT is feared to lead to an increased abortion rate and to social exclusion of people with a disability. Moreover, next‐generation sequencing technologies allow for a future expansion of the scope of NIPT. Some people are concerned that NIPT may come to include trivial conditions or findings that are difficult to interpret.^5^Kelly, S. E., & Farrimond, H. R. (2012). Non‐invasive prenatal genetic testing: A study of public attitudes. *Public Health Genomics, 15*(2), 73–81. Prenatal clinics today are already confronted with—sometimes difficult to interpret—incidental findings resulting from the use of next‐generation technologies in NIPT.^6^Nuffield Council on Bioethics, op. cit. note 4, pp. 26–33.


Another frequently mentioned problem is that NIPT may lead to problems for informed decision‐making: NIPT might be considered by pregnant women as ‘just another blood test’^7^Lewis, C., Hill, M., & Chitty, L. S. (2016). A qualitative study looking at informed choice in the context of non‐invasive prenatal testing for aneuploidy. *Prenatal Diagnosis, 36*(9), 875–881. which is easy to conduct and very reliable. Women might routinely accept NIPT as a screening test for trisomy 21, 18 and 13 and may not be prepared for abnormal test results.^8^de Jong, A., Maya, I., & van Lith, J. M. (2015). Prenatal screening: Current practice, new developments, ethical challenges. *Bioethics, 29*(1), 1–8. Besides, it is feared that women would step into what is called a ‘screening trap’.^9^de Jong, A., & de Wert, G. M. (2015). Prenatal screening: An ethical agenda for the near future. *Bioethics, 29*(1), 46–55. This means that NIPT might put women on a pathway to invasive follow‐up diagnostic testing and potentially termination of the pregnancy, while they not have fully assessed the consequences beforehand.

These problems are considered to challenge the ‘informedness’ of NIPT‐related decisions and consequently to undermine the aim of reproductive autonomy.^10^Dondorp et al., op. cit. note 2, p. 1440. Counselling is the generally preferred instrument to promote informed decisions and includes providing information and decision‐making support.^11^Meiser, B., Irle, J., Lobb, E., & Barlow‐Stewart, K. (2008). Assessment of the content and process of genetic counseling: A critical review of empirical studies. *Journal of Genetic Counseling, 17*(5), 434–451. How can counselling be used to counter some of the ethical and practical problems for informed consent raised by the introduction of NIPT? What should be the focus of counselling, and how can women best be supported in decision‐making for or against first‐trimester prenatal screening?

We first discuss the aim of prenatal screening (reproductive autonomy), the definition of informed consent and its operationalization in counselling. We offer arguments for a renewed recognition and use of the term informed consent—rather than informed choice—in ethical discussions of prenatal screening, and a different understanding of what it means to give or ask for informed consent for first‐trimester screening.

## 2 THE AIM OF PRENATAL SCREENING: PROMOTING REPRODUCTIVE AUTONOMY

The aim of prenatal screening programmes is formulated as promoting reproductive autonomy.^12^Dondorp et al., op. cit. note 2, p. 1440. By explicitly stating this aim, health care systems try to make clear that prenatal screening is different from other forms of screening in the public health context, such as breast or cervical cancer screening, the aims of which are the (secondary) prevention of disease or the promotion of health.^13^Holland et al., op cit. note 1, p. 2. It would be problematic for prenatal screening programmes to be aimed at prevention, for this implies that the birth of affected children ought to be avoided. If that were so, states or healthcare systems might appear to be promoting or encouraging abortion in case of genetic disorders. Abortion would turn into a (eugenic) public health instrument.^14^Dondorp et al., op. cit. note 2, p. 1440. Also, it would carry the discriminatory message that children with the conditions screened for should not be born and their lives are worth less than those of citizens without genetic conditions. Thirdly, it might put pressure on women to terminate the pregnancy of an affected foetus.^15^de Jong et al., op cit. note 9, pp. 48–49; Dondorp et al., op. cit. note 2, p. 1440. Pressure is precisely what should ideally be avoided in decision‐making with regard to NIPT: women must be free to decide whether or not to take part in screening, and whether or not to terminate a pregnancy because of detected abnormalities. To distance prenatal screening from these problems, its aim is formulated as the provision of health‐related information about the foetus in order to offer courses of action to pregnant women and couples in case of a foetal abnormality, or the promotion of reproductive autonomy.^16^Ibid: 48. This means that the decision to reject prenatal screening, too, is and should be part of reproductive autonomy, in recognition of ‘patients’ individual right[s] to decide whether or not they wish to receive testing and then to make reproductive choices based on test results’.^17^Benn, P. A., & Chapman, A. R. (2010). Ethical challenges in providing noninvasive prenatal diagnosis. *Current Opinion in Obstetrics and Gynecology, 22*(2), 128–134.


## 3 INFORMED CONSENT IN THE CONTEXT OF NIPT

The aim of prenatal screening is operationalized through informed consent. Through the instrument of informed consent, healthcare professionals seek to ensure that women make autonomous decisions for or against a screening offer.^18^Bunnik, E. M., Jong, A., Nijsingh, N., & Wert, G. M. W. R. (2013). The new genetics and informed consent: Differentiating choice to preserve autonomy. *Bioethics, 27*(6), 348–355; Walker, T. (2013). Respecting autonomy without disclosing information. *Bioethics, 27*(7), 388–394. According to the seminal theory of informed consent by Faden and Beauchamp, informed consent is given "if a patient or a subject with (1) substantial understanding and (2) in substantial absence of control by others (3) intentionally (4) authorizes a professional (to do I)".^19^Faden, R. R., & Beauchamp, T. L. (1986). *A history and theory of informed consent*. UK: Oxford University Press. Firstly, a decision whether or not to take part in screening should be based on ‘substantial understanding’. This implies that women should be informed about characteristics of the tested condition, potential risks and benefits of the test and implications of possible test outcomes.^20^Marteau, T. M., & Dormandy, E. (2001). Facilitating informed choice in prenatal testing: How well are we doing? *American Journal of Medical Genetics, 106*(3), 185–190. Secondly, women should be free to make a voluntary decision about screening and not be coerced or pressurized by others. Thirdly, women should have the capacity to consent. Most women do, and healthcare professionals are expected to presume that all patients are decisionally competent to decide unless they have reason for doubt. Traditionally, someone is believed to have the capacity to consent when she demonstrates the following four competencies: understanding of relevant information, reasoning based on this information, appreciating her situation and the consequences of her choice, and communicating a choice.^21^Appelbaum, P. S., & Grisso, T. (1988). Assessing patients' capacities to consent to treatment. *New England Journal of Medicine, 319*(25), 1635–1638. Fourthly and finally, the woman must in fact make a choice.

It is noteworthy that in the field of prenatal screening the term ‘informed choice’ is frequently used instead of informed consent, which is ubiquitous in medical ethics and medical practice generally.^22^Hewison, J., & Bryant, L. (2009). Informed consent: What should we be doing? In L. Chitty, S. Kehoe & T. Homfray (Eds.), *Reproductive genetics* (pp. 205–216). UK: Cambridge University Press. In one dominant model, ‘informed choice’ is defined as ‘one that is based on relevant knowledge, consistent with decision‐maker’s values and behaviourally implemented’.^23^Marteau, T. M., Dormandy, E., & Michie, S. (2001). A measure of informed choice. *Health Expectations, 4*(2), 99–108. In the context of NIPT, informed choice is achieved when a woman has sufficient knowledge and either a positive attitude towards undergoing a test while opting for screening, or a negative attitude while refusing screening.

One of the rationales offered for preference of the term informed choice is that it distances prenatal screening programmes from unwanted eugenic associations.^24^Hewison & Bryant, op. cit. note 22, p. 205. Another rationale is that informed choice suggests that decision‐making is less active than in informed consent, and that informed consent requires a more elaborate discussion with a health professional.^25^Jepson, R., Hewison, J., Thompson, A., & Weller, D. (2005). How should we measure informed choice? The case of cancer screening. *Journal of Medical Ethics, 31*(4), 192–196. Also, it is claimed that ‘informed consent is not explicitly concerned with the understanding of those not consenting’.^26^Marteau, T. M. (2009). Informed choice: A construct in search of a name. In A. Edwards & G. Elwyn (Eds.), *Shared decision‐making in health care: Achieving evidence‐based patient choice*. (pp. 87–94). UK: Oxford University Press. Informed consent would suggest that patients should accept the option that is proposed or preferred by the healthcare professional. The withholding of consent to this preferred option might be considered ill‐advised or irrational. By using the term informed *choice* in lieu of informed *consent*, it is emphasized that accepting and rejecting of prenatal screening are evaluated as equally valuable options. Both the choice to accept and the choice to reject prenatal screening are an expression of reproductive autonomy.^27^Deans, Z., & Newson, A. J. (2011). Should non‐invasiveness change informed consent procedures for prenatal diagnosis? *Health Care Analysis, 19*(2), 122–132. Finally, it has been suggested that informed consent ‘is not explicitly concerned with the consenting individual’s values’ while informed choice includes someone’s values reflected in attitudes.^28^Marteau, op. cit. note 26, p. 89.


Also, in the literature on prenatal screening, the term ‘informed decision‐making’ is being used. Informed decision‐making often refers to the pre‐decisional process, ‘the process of arriving at a decision’^29^Elwyn, G., & Miron‐Shatz, T. (2010). Deliberation before determination: The definition and evaluation of good decision making. *Health Expectations, 13*(2), 139–147. and includes a process of deliberation and of weighing of pros and cons,^30^van den Berg, M., Timmermans, D. R. M., ten Kate, L. P., van Vugt, J. M. G., & van der Wal, G. (2006). Informed decision making in the context of prenatal screening. *Patient Education and Counseling, 63*(1–2), 110–117. while informed choice refers to the decision itself for or against a screening offer.

We contend that there is no ethical need for the use of the terms informed choice or informed decision‐making in the context of prenatal screening. Traditional notions of informed consent encompass the criterion of voluntariness, and thus forestall concerns related to a lack of opportunity to withhold consent or related to state‐enforced eugenics. They imply that patients (or pregnant women) understand relevant information about the proposed (or offered) screening test, and that this may require elaborate discussion with a healthcare professional. Also, when a woman is reasoning based on relevant information or appreciating her situation and the consequences of her choice, she is deliberating and evaluating.

As a complement to their ‘autonomous authorisation’ model, Faden and Beauchamp propose a condition of authenticity:

An authenticity condition would require actions to be consistent with a person's reflectively accepted values and behaviour in order to be autonomous. Authenticity in this usage requires that actions faithfully represent the values, attitudes, motivations, and life plans that the individual personally accepts upon due consideration of the way he or she wishes to live.^31^Faden & Beauchamp, op. cit. note 19, p. 263.

With this condition, the traditional model of informed consent incorporates the consenting individual’s values and attitudes. Ultimately, in this ‘autonomous authorisation plus authenticity’ model, informed consent in the context of prenatal screening would require women’s choices to be deliberate and consistent with their values as reflected in their attitudes. Thus, the rationales offered in the literature for preferring the term informed choice (or decision‐making) over the term informed consent, do not hold.

Besides, a rehabilitation of the notion of informed consent in the context of prenatal screening may offer the added benefit of embedding it in the broader basis of existing ethical literature concerning the principle of respect for autonomy, which plays an especially important role in ethical discussions of NIPT as its main aim.

## 4 LIMITATIONS OF CURRENT MODELS FOR ‘INFORMED CONSENT’

Given the aim of prenatal screening, to evaluate the success of screening programmes for aneuploidies including pre‐test counselling, the *informedness* of women’s decisions for or against screening must be assessed, rather than uptake or detection rates.^32^Dondorp et al., op. cit. note 2, p. 1440. Various measures of informed consent and informed choice have been developed in the past to measure the ‘informedness’ of women’s choices with regard to screening offers.^33^Piechan, J. L., Hines, K. A., Koller, D. L., Stone, K., Quaid, K., Torres‐Martinez, W., … Cook, L. (2016). NIPT and informed consent: An assessment of patient understanding of a negative NIPT result. *Journal of Genetic Counseling, 25*(5), 1127–1137; Constantine, M. L., Allyse, M., Wall, M., Vries, R. D., & Rockwood, T. H. (2013). Imperfect informed consent for prenatal screening: Lessons from the quad screen. *Clinical Ethics, 9*(1), 17–27; Marteau, T. M., Dormandy, E., & Michie, S. (2001). A measure of informed choice. *Health Expectations, 4*(2), 99–108.


The knowledge component of these models, however, is problematic for NIPT. Firstly, the necessity of knowledge might get too little attention amongst women because, as said, the procedural ease of NIPT could hinder women’s understanding that they have to provide informed consent for first‐trimester prenatal screening, leading to routine acceptance of the test.^34^Deans, Z., Hill, M., Chitty, L. S., & Lewis, C. (2013). Non‐invasive prenatal testing for single gene disorders: Exploring the ethics. *European Journal of Human Genetics, 21*(7), 713–718. Furthermore the next‐generation sequencing technologies used for the test and its possible outcomes—trisomy 21, 13 and 18, and incidental findings—are increasingly complex. There are concerns that women may lack understanding of relevant information about its aim, procedures, possible outcomes and consequences. Also, it may not be possible to redress these concerns by having healthcare professionals provide more and more—written and verbal—information to pregnant women. In fact, the provision of a lot of medical‐technical information during pre‐test counselling may overwhelm women and cause ‘information overload’, which may hinder them in becoming aware of what prenatal screening might mean for them.^35^Dondorp, W.J., Page‐Christiaens, G.C.M.L., de Wert, G.M.W.R. (2016). Genomic futures of prenatal screening: ethical reflection. *Clinical Genetics*, *89*(5), 531–538. http://dx.doi.org/10.1111/cge.12640. When measurement scales focused on information and knowledge are being used to assess the quality of informed consent, such assessments are likely to result in high percentages of ‘uninformed’ decisions. But is that to say that women have not given valid informed consent for screening, or that their decisions were not autonomous?

Providing or ‘disclosing’ information may not be a primary requirement for informed consent in the context of prenatal screening. Manson and O’Neill have pointed out the complexities of the disclosure or what they call the ‘conduit’ of information in the context of consent. Information, for instance, is ‘inferentially fertile’^36^Manson, N. C., & O’Neill, O. (2007). *Rethinking informed consent in bioethics*. New York, NY: Cambridge University Press.: when a pregnant woman receives a bit of information about a test, she may consciously or unconsciously go on to make a range of inferences about the test, which may or may not overlap with the counsellor’s understanding of the test and may or may not be correct or relevant. Moreover, when she enters the counselling session, she may have already made her decision about participation in screening.^37^van den Berg, M., Timmermans, D. R. M., Knol, D. L., van Eijk, J. T. M., de Smit, D. J., van Vugt, J. M. G., & van der Wal, G. (2008). Understanding pregnant women's decision making concerning prenatal screening. *Health Psychology, 27*(4), 430–437. She may have gathered her information from various types of sources (e.g. magazines, acquaintances, social media). Thus, when she consents, she may consent to something (slightly) different than that which is envisioned and disclosed to her by the counsellor.

Pre‐test counselling should therefore not focus on the knowledge component of informed consent, but on supporting pregnant women and their partners in making personal, value‐consistent decisions about prenatal screening. This is how reproductive autonomy is best served. Offering decision‐making support can at the same time be used to counter the problem of routine acceptance of prenatal screening: although NIPT is not a diagnostic test, and any abnormal results must be confirmed through invasive follow‐up testing, it is much more sensitive and specific than previous technologies. It further requires only a single blood draw. As women may thus have fewer reasons to refuse screening, they may accept it automatically, without full consideration. Focusing on personal decision‐making might help women to make a personal decision about prenatal screening.

Screening is offered to help women to plan their lives according to their values—if they want to, with use of prenatal screening. Women therefore should make considered decisions for or against first‐trimester screening, for it may have great impact on their lives. The decision to take part or not to take part in screening should be informed but above all authentic. To respect women’s autonomy and enable them to decide about prenatal screening according to their personal values they should be enabled to think about the question why they would want to know whether their baby has a genetic disorder. Women should be prompted to think about whether they want to have the options (termination or preparation in case of a genetic disorder) that prenatal screening provides them in order to plan their lives. This is in line with the notion that informed consent includes a more active decision‐making process than informed choice.^38^Jepson et al., op. cit. note 25, p. 193. Reproductive autonomy not only involves sufficient knowledge as argued by previous authors but also ‘involves (…) encouraging self‐reflection to act in accordance with broader life goals’,^39^Newson, A. J. (2016). Why information and choice won't solve all of NIPT's ethical problems. Bionews. Retrieved from https://www.bionews.org.uk/page_95667
 which emphasizes autonomous decision‐making. This aim is more in line with the definition of informed consent including the authenticity requirement, as it focuses on self‐determination and the broader ideal of planning one’s life according to one’s values.

## 5 A THREE‐STEP COUNSELLING MODEL

To reach an authentic choice according to someone’s life plan requires a restructuring of the current approach to counselling, and requires primarily a dialogue about the pregnant woman’s or couple’s values, instead of providing ‘value free’^40^Vanstone, M., Kinsella, E. A., & Nisker, J. (2012). Information‐sharing to promote informed choice in prenatal screening in the spirit of the SOGC clinical practice guideline: A proposal for an alternative model. *Journal of Obstetrics and Gynaecology Canada, 34*(3), 269–275. medical‐technical information as is suggested by several professional committees.^41^Cartier, L., Murphy‐Kaulbeck, L., Wilson, R. D., Audibert, F., Brock, J.‐A., Carroll, J., … Pastuck, M. (2012). Counselling considerations for prenatal genetic screening. *Journal of Obstetrics and Gynaecology Canada, 34*(5), 489–493; Devers, P. L., Cronister, A., Ormond, K. E., Facio, F., Brasington, C. K., & Flodman, P. (2013). Noninvasive prenatal testing/noninvasive prenatal diagnosis: The position of the national society of genetic counselors. *Journal of Genetic Counseling, 22*(3), 291–295.


**FIGURE 1:**
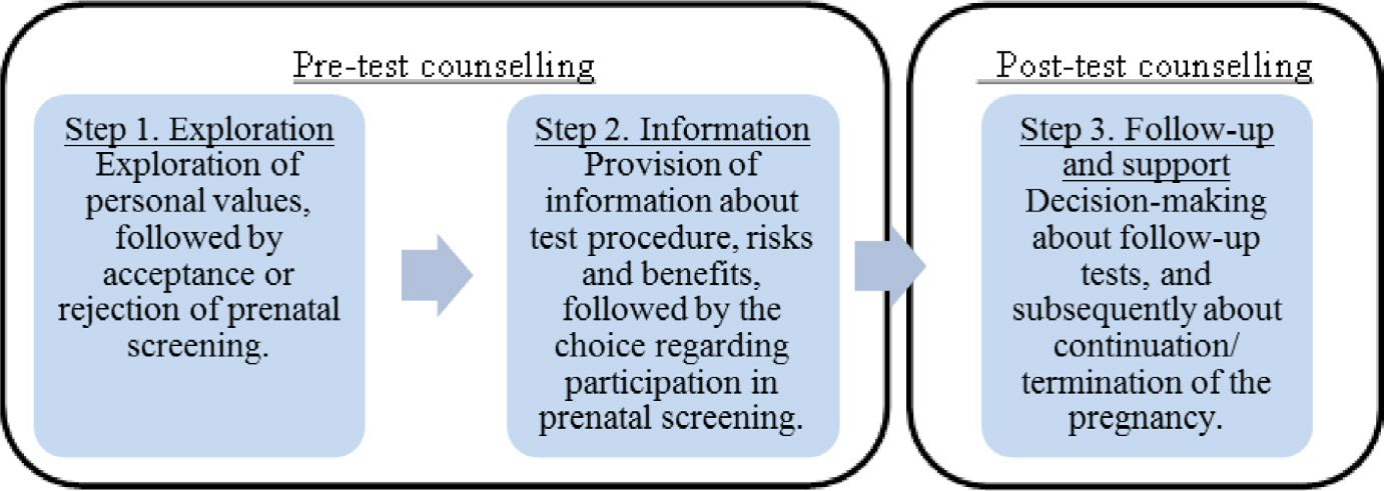
A three‐step counselling model.

We propose a re‐focusing of pre‐ and post‐test counselling and a re‐envisioning of the decision‐making process, consisting of three central decision moments for women and their partners (Figure 1). These three decision moments are derived from the current counselling practice in the Netherlands, in which pregnant women are already presented with three decision moments. In the Netherlands pregnant women first receive an ‘information offer’. With this offer, a woman is asked whether she would want to receive information about prenatal screening at all. When a woman declines, the counsellor will explore her motivation and will not inform her any further about first‐trimester prenatal screening options.^42^Rijksinstituut voor Volksgezondheid en Milieu. (2018). *Kwaliteitseisen counseling prenatale screening versie 10*. Den Haag, the Netherlands: Rijksinstituut voor Volksgezondheid en Millieu. The information offer is meant to promote the moral *right not to know* about the options of prenatal screening for foetal aneuploidies, in order to stress the fact that this screening is not mandatory.^43^Gezondheidsraad. (2016). *Juridische aspecten van prenatale screening: Achtergronddocument bij prenatale screening*. Den Haag, the Netherlands: Gezondheidsraad.


Critics of the information offer suggest that it is not possible to make an informed choice to decline screening when one does not know about the options for prenatal screening. This criticism touches upon a realistic problem, but we think that the solution is not to provide complete information in this first step. Instead, the health professional first should explore women’s motivations and related values to determine whether the declination is either the result of an autonomous decision or on misunderstanding of prenatal screening.

### 5.1 Step 1: Exploration

The first decision moment of our proposed counselling model focuses on women’s personal attitudes towards prenatal screening and its meaning to their life planning, instead of providing medical‐technical information. The main goal of this first step is that health professionals will explore women’s values, discussing with them why they do or do not want to know about genetic disorders at this stage of pregnancy. This might enable women to make their values explicit in the context of this decision. We acknowledge that in this step women might want some information about prenatal screening, for example to imagine what possible results might mean to them. But foremost, in this first step it must become clear to women that opting for—or against—prenatal screening is a free and personal choice: it should focus on promoting choice awareness. More than the information offer, this first step might infringe upon the presumed right not to know about screening options. This first step does not replace the information offer, because in this step it is about accepting or declining the screening offer, not an information offer. An information offer could take place beforehand, but might entail the same exploration questions to find out whether women or couples have deliberated about their decision.

### 5.2 Step 2: Information

The second step in the counselling is that, when women would like to have prenatal screening, they will receive information about the test, its procedures, its possible outcomes and the consequences thereof, and risks and benefits. At this stage medical‐technical information becomes more important and provides women the option to compare this information with their values. Information provision can be done through multiple modalities, including written materials, video materials, individual and/or group‐based face‐to‐face discussions with healthcare professionals, according to women’s personal needs, to ensure that key information on the (increasingly) complex test is conveyed. In this step it should again be stressed that women are free to withdraw from taking part in screening.

In the current Dutch practice of offering NIPT, wherein women can choose to learn about incidental findings, the question arises as to whether women need to know everything about the abnormalities included in the test, before they opt for screening, or whether they could wait to receive a full explanation of the implications of detected abnormalities when it turns out that one has been detected. We suggest that in order to make an informed choice, in the second decision moment women do not necessarily need to know medical‐technical information about the test, such as the percentage of women that has a low risk based on first‐trimester screening or which follow‐up tests are available beforehand. They primarily should understand that first‐trimester prenatal screening may yield information about serious diseases for which often no treatment is available. They should know that this may be a reason for women or couples to consider termination of an affected pregnancy, and should consider whether or not they wish to make use of the possibility of obtaining such information about their foetus. However, women’s preferences, concerning which information is provided, how much and in what way, might differ. To design the second step, a tiered‐layered‐staged model for informed consent, which has been proposed in the context of genomic testing, might provide direction, proposing a choice between specified categories of diseases.^44^Bunnik, E. M., Janssens, A. C. J., & Schermer, M. H. (2013). A tiered‐layered‐staged model for informed consent in personal genome testing. *European Journal of Human Genetics, 21*(6), 596–601. In the context of prenatal screening and pre‐test counselling, categories of incidental findings can be based on characteristics of abnormalities, for example pathogenic for the foetus, variants of unknown clinical significance, benign findings and incidental findings, as proposed for diagnostic genetic tests.^45^Srebniak, M. I., Diderich, K. E. M., Govaerts, L. C. P., Joosten, M., Riedijk, S., Galjaard, R. J. H., & Van Opstal, D. (2014). Types of array findings detectable in cytogenetic diagnosis: A proposal for a generic classification. *European Journal of Human Genetics, 22*(7), 856–858. Based on these categories women and couples can be informed about possible outcomes according to their needs, to make a personal informed decision about prenatal screening. Furthermore, in the second step, information about the prenatal test and its outcomes could be presented in a layered fashion, offering more detailed information (written materials, websites, group information meetings) to women on request, in order to keep the first layer of information (offered during the face‐to‐face counselling discussion with the healthcare professional) limited and focused on key messages, preventing information overload. Besides, information provision could be spread over time to promote elaboration about the information and reflection on it,^46^Bunnik et al., op. cit. note 44, p. 599. although in the context of prenatal screening counsellors should take account of the fact that during a pregnancy, the time of having courses of action, including the possibility to terminate the pregnancy, is limited and thus the time to reflect on information is limited.

Ultimately, in step two, women should again be encouraged to reflect on the information provided based on their personal values. Therefore, the information given in step two should foremost support value‐consistency, and not be aimed at providing as much objective technical‐medical information as possible.

### 5.3 Step 3: Follow‐up and support

The third step takes place when women receive an abnormal test result. This step does not differ from the current practice after receiving an abnormal result from prenatal screening. Women will receive post‐test genetic counselling from one or more relevant professionals, in most cases a clinical geneticist, about the detected abnormality, its prognosis and possible courses of action. After considering this information women and their partners should obtain information about follow‐up tests including amniocentesis or chorionic villus sampling, the consequences of carrying the pregnancy to term or terminating the pregnancy. They should be free to decide whether or not to opt for follow‐up tests and termination or continuation of the pregnancy and receive professional support during their decision‐making.

This three‐stage choice process covers the problem that NIPT might cause for informed consent. It moves towards resolving the problems of routine uptake of prenatal screening by emphasizing the personal‐choice aspect, focusing on women’s or couples’ personal values. This stepwise counselling model, including the layered information provision might also be applicable to other types of prenatal screening like the 20‐week ultrasound scan, and also to other types of genetic testing such as, for example for parents with a known family history of a genetic history. Furthermore, this restructuring of pre‐test counselling could address the concern that reproductive autonomy could be hindered by future expansions in conditions screened for with prenatal screening tests. It is feared that therewith NIPT will involve too much information about many abnormalities, which might cause an information overload for women during pre‐test counselling. Women might not understand what a broad NIPT might disclose, hindering them from giving informed consent about whether or not to participate in screening.^47^Dondorp et al., op. cit. note 2, p. 1444.


But for the first of the three decision moments, the width of the scope and the technicalities of the test are of less importance. The most important question is whether women want the options prenatal screening might provide to them, including preparation and termination of pregnancy in case of a genetic disorder.

## 6 THE THREE‐STEP MODEL IN PRACTICE

Our proposal to change the focus of pre‐test counselling from information provision towards elaborating women’s values is not fully new. Studies amongst pregnant women found that not only information about a test but also personal circumstances^48^Lewis et al., op. cit. note 7, p. 877. and ethical beliefs influence their decision. Furthermore women want to have time to deliberate,^49^Lewis, C., Silcock, C., & Chitty, L. S. (2013). Non‐invasive prenatal testing for Down's syndrome: Pregnant women's views and likely uptake. *Public Health Genomics, 16*(5), 223–232. and prefer a form of advice besides non‐directive health education.^50^Martin, L., Dulmen, S. V., Spelten, E., Jonge, A. D., Cock, P. D., & Hutton, E. (2013). Prenatal counseling for congenital anomaly tests: Parental preferences and perceptions of midwife performance. *Prenatal Diagnosis, 33*(4), 341–353. Professionals indicated that they should ‘trigger women to think’^51^Kater‐Kuipers, A., Bunnik, E. M., de Beaufort, I. D., & Galjaard, R. J. H. (2018). Limits to the scope of non‐invasive prenatal testing (NIPT): An analysis of the international ethical framework for prenatal screening and an interview study with Dutch professionals. *BMC Pregnancy and Childbirth, 18*(1), 409. and midwives thought that it is important to ask exploring questions that make women think.^52^Martin, L., Hutton, E. K., Spelten, E. R., Gitsels‐van der Wal, J. T., & van Dulmen, S. (2014). Midwives' views on appropriate antenatal counselling for congenital anomaly tests: Do they match clients' preferences? *Midwifery, 30*(6), 600–609. However, they indicated that they experience a lack of time to ask them. The lack of time could be solved with decision‐aids, which can help women to prepare for the counselling and already obtain information about prenatal screening, or to resume what is discussed in the counselling, facilitating a staged process. Although some women might wish to receive information about prenatal screening in a separate visit, steps one and two of the counselling model could take place in a single visit. Nevertheless, two separate counselling moments do not necessarily demand very many additional resources because it often can take place in visits wherein other topics are discussed and measurements are done. But, as professionals have already underlined, to provide women with time to consider, the prenatal test should not take place at the same visit as the pre‐test counselling.^53^Tamminga, S., van Schendel, R. V., Rommers, W., Bilardo, C. M., Pajkrt, E., Dondorp, W. J., … Henneman, L. (2015). Changing to NIPT as a first‐tier screening test and future perspectives: Opinions of health professionals. *Prenatal Diagnosis, 35*(13), 1316–1323.


The three‐step counselling model might fulfil women’s needs of support in making a decision according to their beliefs and help counsellors to facilitate reflection on women’s choices for or against prenatal screening. Furthermore, it might protect those women who are less able to understand information and formulate their personal values and promote their reproductive autonomy, corresponding to what is stated by O’Neill: ‘Informed consent procedures protect choices that are timid, conventional and lacking in individual autonomy (variously conceived) just as much as the protect choices that are self‐assertive’.^54^O’Neill, O. (2003). Some limits of informed consent. *Journal of Medical Ethics, 29*(1), 4–7. In the Dutch context, it may help to avoid the moral discomfort experienced by professionals when they do not provide any information at all to those who decline the information offer. The present article shows that there are also ethical arguments for a revised approach of pre‐test counselling for prenatal screening, which should focus on personal decision‐making.

Finally, as reproductive autonomy also includes relational aspects,^55^Newson, op. cit. note 39. enabling women to give informed consent and attaining the aim of the prenatal screening programme, is not only the responsibility of counsellors. As argued elsewhere, also the context in which a decision is made matters.^56^Kater‐Kuipers Adriana, de Beaufort Inez, D., Galjaard Robert‐Jan, H., Bunnik Eline, M. (2018). Ethics of routine: a critical analysis of the concept of ‘routinisation’ in prenatal screening. *Journal of Medical Ethics*, *44*(9), 626–631. http://dx.doi.org/10.1136/medethics‐2017‐104729. Women or couples should have the feeling that accepting or rejecting prenatal screening are equally valuable options. This is not only established by counselling but also by the broader societal context, in which provision of care and support should be in place for those who choose to continue a pregnancy when it is known that the child born will have a disability, as well as for those who choose to terminate the pregnancy.

## 7 CONCLUSION

The introduction of NIPT is associated with several ethical problems including negative consequences for informed consent. Because of its procedural ease, NIPT is believed to hinder women’s understanding that they have to personally decide about a first‐trimester prenatal screening offer. Furthermore, the potential for future expansion of NIPT might pose challenges for sufficient information provision. The current way of counselling focuses on the non‐directive provision of practical and medical‐technical information about the test, and may not be equipped to counter these problems. Informed consent in prenatal screening should be characterized as the decision to participate or not participate in screening, based on an understanding that screening may yield information about serious disorders in the foetus, which may be a reason for women and their partners to consider termination of the pregnancy. In our view, having knowledge about the test itself, its possible outcomes and the consequences thereof may be conducive to the informed consent process for some women, but it is not of central importance to all women.

We have proposed a three‐step counselling model, in which three decision moments are distinguished and recognized as different types of decisions, for which different types of counselling should be offered to women and their partners. The primary decision should focus on the values concerning obtaining knowledge about whether the baby has a genetic disorder and the courses of option this knowledge provides. The second step involves layered information provision about the test and the final decision to test or not test, adapted to women’s personal informational need. In case of an abnormal test result, in a third step, women will need to decide about follow‐up tests and the continuation of their pregnancy. We have argued that achieve the aim of prenatal screening not necessarily lies in having sufficient knowledge, but in making a personal choice, according to one’s life plan.

## CONFLICT OF INTEREST

The authors declare no conflict of interest.

